# Development and evaluation of a core genome multilocus typing scheme for whole-genome sequence-based typing of *Acinetobacter baumannii*

**DOI:** 10.1371/journal.pone.0179228

**Published:** 2017-06-08

**Authors:** Paul G. Higgins, Karola Prior, Dag Harmsen, Harald Seifert

**Affiliations:** 1 Institute for Medical Microbiology, Immunology and Hygiene, University of Cologne, Cologne, Germany; 2 German Centre for Infection Research (DZIF), partner site Bonn-Cologne, Cologne, Germany; 3 Department for Periodontology and Restorative Dentistry, University Hospital Muenster, Muenster, Germany; Tianjin University, CHINA

## Abstract

We have employed whole genome sequencing to define and evaluate a core genome multilocus sequence typing (cgMLST) scheme for *Acinetobacter baumannii*. To define a core genome we downloaded a total of 1,573 putative *A*. *baumannii* genomes from NCBI as well as representative isolates belonging to the eight previously described international *A*. *baumannii* clonal lineages. The core genome was then employed against a total of fifty-three carbapenem-resistant *A*. *baumannii* isolates that were previously typed by PFGE and linked to hospital outbreaks in eight German cities. We defined a core genome of 2,390 genes of which an average 98.4% were called successfully from 1,339 *A*. *baumannii* genomes, while *Acinetobacter nosocomialis*, *Acinetobacter pittii*, and *Acinetobacter calcoaceticus* resulted in 71.2%, 33.3%, and 23.2% good targets, respectively. When tested against the previously identified outbreak strains, we found good correlation between PFGE and cgMLST clustering, with 0–8 allelic differences within a pulsotype, and 40–2,166 differences between pulsotypes. The highest number of allelic differences was between the isolates representing the international clones. This typing scheme was highly discriminatory and identified separate *A*. *baumannii* outbreaks. Moreover, because a standardised cgMLST nomenclature is used, the system will allow inter-laboratory exchange of data.

## Introduction

*Acinetobacter baumannii* is now a recognised serious nosocomial pathogen and is isolated frequently particularly in intensive care unit settings where it is a cause of serious infections such as ventilator-associated pneumonia, wound and bloodstream infections [1]. It affects mainly severely debilitated patients and is typically selected by prior antimicrobial therapy [2]. *A*. *baumannii* shares several characteristics with methicillin-resistant *Staphylococcus aureus* (MRSA) such as multidrug resistance and long-term survival on inanimate surfaces such as computer keyboards, pillows, curtains and other dry surfaces. [2–4]. This longevity contributes to hospital outbreaks and the clonal spread of isolates, and it facilitates person-to-person transmission and environmental contamination. Strict adherence to infection control measures and sometimes even the closure of wards is required for the control of outbreaks [5].

Many typing methods have been used to investigate outbreaks of *A*. *baumannii*, and its clonal population structure is now well-established. Molecular typing of isolates obtained from various locations in the EU has shown the existence of three distinct clonal lineages that were termed pan-European clonal complexes I, II and III [6,7]. Rep-PCR (DiversiLab) has shown that these lineages are no longer restricted to Europe and that there exist at least eight distinct international clonal lineages that are particularly associated with carbapenem-resistance [8]. Furthermore, multilocus sequence typing (MLST) has corroborated these data [9–11].

Pulsed-field gel electrophoresis (PFGE) is still considered the gold standard for outbreak analysis and although the method has been standardized, there is still the problem of reproducibility and portability of these data outside of reference laboratories [12–14]. Similarly, with rep-PCR we demonstrated that overlaying data generated from different centres is not reliable [15]. Perhaps the only truly portable established method is MLST, but this lacks the resolution for outbreak investigations [16].

With the advent of relatively cheap whole genome sequencing (WGS), there is now the possibility of comparing whole genomes and not relying on a few loci for typing purposes. There have been several studies that have addressed this by either comparing isolates on single nucleotide variants (SNVs) or genome-wide gene-by-gene allelic profiling, which is now termed core genome MLST (cgMLST) [17–20]. One barrier to the ready adoption of WGS for routine typing is the data analysis, which can be difficult for the non-bioinformatician. Therefore the development of user-friendly software is expected to greatly enhance the adoption of WGS [21,22].

The objective of this study was to establish a cgMLST scheme for *A*. *baumannii* that can form the basis of a standardised nomenclature for typing this organism. To enable this, we first defined a core genome gene set that represented the genetic diversity of *A*. *baumannii* based on well-characterised reference strains from our collection and those available online. We then challenged the scheme against data available from NCBI. Well-defined outbreaks were sequenced and analysed to determine the scheme’s suitability for outbreak investigations.

## Material and methods

### Bacterial isolates and DNA extraction

For scheme calibration, a total of 53 carbapenem-susceptible and -resistant *A*. *baumannii* isolates from well-described hospital outbreaks occurring throughout Germany were used, some of which have been the subject of a previous report (Table 1) [23]. The isolates had previously been assigned to international clones (IC) 1, 2, 4, 7, and 8 using rep-PCR, and their carbapenem resistance mechanisms determined by PCR [8,11,24]. Two isolates were not considered as clustering with the ICs. For the purpose of this study, an outbreak was defined as two or more isolates from a given hospital and from patients that were linked in time and space that had highly similar or identical PFGE patterns as had been determined previously. PFGE subtypes were defined as having 1–3 band differences. In some cases, there was more than one circulating clone in the same hospital. Isolates were routinely grown on blood agar and after overnight growth in LB liquid media, DNA was extracted using the MagAttract HMW DNA isolation kit following the manufacturer’s instructions (Qiagen, Hilden, Germany) and quantified using the Qubit dsDNA BR assay (Fisher Scientific GmbH, Schwerte, Germany).

**Table 1:** *A*. *baumannii* strains used for *A*. *baumannii* cgMLST cluster calibration.

Cluster	Strain	Hospital	City of Isolation	Year of Isolation	PFGE Type^a^	ST (‘Oxford’/ ‘Pasteur’)	Clonal Lineage^b^	BAPS Partition	Carbapenem-resistance Determinant	Coverage (Assembled)	Contig Count (Assembled)	% good cgMLST Genes	ENA Accession
I	UKK_0008	3	Berlin	2006	2-A1	436/2	IC2	2	OXA-23	108	112	99.8	ERS1042999
I	UKK_0012	3	Berlin	2006	2-A1	436/2	IC2	2	OXA-23	98	107	99.8	ERS1043003
I	UKK_0016	3	Berlin	2006	2-A1	436/2	IC2	2	OXA-23	99	160	99.7	ERS1043004
I	UKK_0024	3	Berlin	2006	not done	436/2	IC2	2	OXA-23	114	130	99.7	ERS1043005
I	UKK_0031	3	Berlin	2006	2-A1	436/2	IC2	2	OXA-23	116	102	99.8	ERS1043006
I	UKK_0038	3	Berlin	2006	2-A2	436/2	IC2	2	OXA-23	109	99	99.8	ERS1043007
I	UKK_0047	3	Berlin	2006	2-A1	436/2	IC2	2	OXA-23	94	145	99.8	ERS1043008
I	UKK_0052	3	Berlin	2006	2-A1	436/2	IC2	2	OXA-23	74	123	99.8	ERS1043009
II	UKK_0252	8	Leverkusen	2011	4-A	218/2	IC2	2	OXA-23	56	71	99.8	ERS1043096
II	UKK_0254	8	Leverkusen	2011	4-A	218/2	IC2	2	OXA-23	104	121	99.7	ERS1043128
II	UKK_0260	8	Leverkusen	2011	4-A	218/2	IC2	2	OXA-23	116	178	99.8	ERS1043151
II	UKK_0263	8	Leverkusen	2011	4-A	218/2	IC2	2	OXA-23	37	75	99.7	ERS1043152
II	UKK_0266	8	Leverkusen	2012	4-A	218/2	IC2	2	OXA-23	92	67	99.9	ERS1043178
III	UKK_0253	8	Leverkusen	2011	4-B	195/2	IC2	2	OXA-23	118	157	99.7	ERS1043117
III	UKK_0255	8	Leverkusen	2011	4-B	195/2	IC2	2	OXA-23	119	108	99.9	ERS1043139
III	UKK_0256	8	Leverkusen	2011	4-B	195/2	IC2	2	OXA-23	119	183	99.7	ERS1043150
III	UKK_0264	8	Leverkusen	2011	4-B	195/2	IC2	2	OXA-23	73	140	99.7	ERS1043177
IV	UKK_0173	5	Cologne	2011	6A	558/2	IC2	2	no	119	120	99.6	ERS1043040
IV	UKK_0174	5	Cologne	2011	6A	558/2	IC2	2	no	113	104	99.6	ERS1043042
IV	UKK_0177	5	Cologne	2011	6A	558/2	IC2	2	no	117	159	99.6	ERS1043043
V	UKK_0178	5	Cologne	2011	6-B	448/2	IC2	admixture	OXA-23	102	178	99.3	ERS1043044
V	UKK_0179	5	Cologne	2011	6-B	448/2	IC2	admixture	OXA-23	79	159	99.2	ERS1043045
V	UKK_0180	5	Cologne	2011	6-B	448/2	IC2	admixture	OXA-23	116	276	99.2	ERS1043057
VI	UKK_0182	6	Cologne	2011	7-A1	448/2	IC2	admixture	OXA-23	123	147	99.3	ERS1043059
VI	UKK_0183	6	Cologne	2011	7-A2	448/2	IC2	admixture	OXA-23	124	156	99.0	ERS1043089
VI	UKK_0185	6	Cologne	2011	7-A1	448/2	IC2	admixture	OXA-23	124	142	99.2	ERS1043090
VI	UKK_0193	6	Cologne	2011	7-A2	448/2	IC2	admixture	OXA-23	122	123	99.2	ERS1043092
VI	UKK_0196	6	Cologne	2011	7-A2	448/2	IC2	admixture	OXA-23	98	153	99.2	ERS1043093
VII	UKK_0056	2	Berlin	2007	1-B	350/2	IC2	2	OXA-58	69	70	99.9	ERS1043010
VII	UKK_0057	2	Berlin	2007	1-B	350/2	IC2	2	OXA-58	67	78	99.9	ERS1043029
VII	UKK_0058	2	Berlin	2007	1-B	350/2	IC2	2	OXA-58	64	75	99.9	ERS1043030
VIII	UKK_0004	3	Berlin	2005	2-B	438/15	IC4	4	OXA-58	116	95	99.4	ERS1042932
VIII	UKK_0005	3	Berlin	2005	2-B	438/15	IC4	4	OXA-58	111	118	99.3	ERS1042933
VIII	UKK_0006	3	Berlin	2005	2-B	438/15	IC4	4	OXA-58	114	142	99.2	ERS1042934
VIII	UKK_0007	3	Berlin	2005	2-B	438/15	IC4	4	OXA-58	116	124	99.1	ERS1042935
IX	UKK_0412	9	Ludwigshafen	2013	8-A	933/94	IC1	not done^d^	OXA-23	117	104	99.1	ERS1043424
IX	UKK_0413	9	Ludwigshafen	2013	8-A	933/94	IC1	not done	OXA-23	57	150	99.2	ERS1043425
IX	UKK_0414	9	Ludwigshafen	2013	8-A	933/94	IC1	not done	OXA-23	104	155	99.1	ERS1043426
IX	UKK_0415	9	Ludwigshafen	2014	8-A	933/94	IC1	1C	OXA-23	101	173	99.1	ERS1043427
X	UKK_0283	1	Aachen	2010	9-A1	229/25	IC7	1A	OXA-23	59	141	99.2	ERS1043179
X	UKK_0284	1	Aachen	2010	9-A1	229/25	IC7	1A	OXA-23	113	80	99.2	ERS1043215
X	UKK_0285	1	Aachen	2011	9-A2	229/25	IC7	1A	OXA-23	104	96	99.2	ERS1043216
X	UKK_0286	1	Aachen	2011	9-A2	229/25	IC7	1A	OXA-23	58	186	99.1	ERS1043217
X	UKK_0290	1	Aachen	2011	not done	229/25	IC7	1A	OXA-23	115	100	98.2	ERS1043218
X	UKK_0295	1	Aachen	2011	not done	229/25	IC7	1A	OXA-23	98	91	99.2	ERS1043219
XI	UKK_0386	4	Bonn	2013	10-A	229/25	IC7	1A	OXA-23	110	125	99.0	ERS1043422
XI	UKK_0409	4	Bonn	2013	10-A	229/25	IC7	1A	OXA-23	93	79	99.1	ERS1043423
XII	UKK_0306	9	Ludwigshafen	2012	8-B1	1118/136	unc^c^	not done	no	58	90	99.0	ERS1043220
XII	UKK_0318	9	Ludwigshafen	2012	8-B2	1118/136	unc	not done	no	95	333	99.0	ERS1043221
XIII	UKK_0333	7	Cologne	2013	11-A	391/157	IC8	1C	OXA-23	68	194	98.8	ERS1043342
XIII	UKK_0334	7	Cologne	2012	11-A	391/157	IC8	1C	OXA-23	59	93	98.8	ERS1043375
XIII	UKK_0368	7	Cologne	2013	11-A	391/157	IC8	1C	OXA-23	53	103	98.7	ERS1043384
XIII	UKK_0369	7	Cologne	2013	11-A	391/157	IC8	1C	OXA-23	87	100	98.7	ERS1043385

^a^PFGE types: The first numeral indicates the hospital, the letter is for pulsotype which is specific to that hospital, and in some cases there is a second numeral for subtype.

^b^Clonal lineage based on rep-PCR clustering.

^c^unc; unclassified as it did not cluster with one of the ICs.

^d^STs above ST920 were not available when BAPS analysis was performed, therefore not included in BAPS partitioning.

### Whole genome sequencing and assembly

Sequencing libraries were prepared using the Nextera XT library prep kit (Illumina GmbH, Munich, Germany) for a 250bp paired-end sequencing run on an Illumina MiSeq sequencer. Samples were sequenced to aim for a minimum 100-fold coverage using Illumina’s recommended standard protocols with dual-index barcoding and rotation of barcodes over time. Sequencing run quality (Q30 and output) had to fulfill the manufacturer’s minimum specifications. The resulting FASTQ files were quality trimmed and assembled *de novo* using the Velvet assembler that is integrated in Ridom SeqSphere^+^ v.3.0 software (Ridom GmbH, Münster, Germany) [25]. Here, reads were trimmed at their 5'- and 3'-ends until an average base quality of 30 was reached in a window of 20 bases, and the assembly was performed with Velvet version 1.1.04 [26] using optimized k-mer size and coverage cutoff values based on the average length of contigs with > 1000 bp.

### BAPS

To determine the overall *A*. *baumannii* species variation, we applied a Bayesian analysis of population structure (BAPS) [27] with the more discriminatory MLST scheme described by Bartual et al [28]. All MLST profiles available as of March 25^th^ 2015 (913 sequence types [ST]) were downloaded from the PubMLST web site (http://pubmlst.org/abaumannii/), all allelic gene sequences per locus were multiple aligned using MUSCLE [29] and finally concatenated for each ST. The BAPS analysis was carried out using the clustering of linked molecular data functionality. Ten runs were performed setting an upper limit of 20 partitions. Admixture analysis was performed using the following parameters: minimum population size considered 5, iterations 50, number of reference individuals simulated from each population 50, number of iterations for each reference individual 10. Those STs that had significant (*P*< 0.05) admixture were not assigned to a partition. The aligned and concatenated ST sequences were used to produce a maximum likelihood (ML) tree using FastTree 2 [30]. Finally, the assignment of sequence types to BAPS partitions was visualized by coloring the nodes (representing the individual STs) of the radial phylogram calculated with FastTree 2 that was drawn by Dendroscope 3 [31]. The largest resulting partition was further subdivided by visual inspection of the phylogram.

### cgMLST target gene definition

To determine the cgMLST gene set, a genome-wide gene-by-gene comparison was performed using the cgMLST target definer (version 1.1) function of the SeqSphere^+^ (Ridom GmbH, Münster, Germany) software with default parameters as described previously [17]. The ACICU strain served as reference genome (NC_010611.1, dated 12 August 2015) [32]. BLAST version 2.2.12 was used for pairwise comparison with the *A*. *baumannii* query genomes (Table 2).

**Table 2:** List of *A*. *baumannii* strains and genomes used for cgMLST *A*. *baumannii* target definition.

Strain	Lineage	BAPS Partition	NCBI Genome Status	ST ‘Oxford’	ST ‘Pasteur’	Avg. Coverage (Assembled)	NCBI/ENA Accession
ACICU (Reference)	IC2	2	Complete Genome	437	2	n/a	NC_010611.1
AYE	IC1	4	Complete Genome	231	1	n/a	NC_010410.1
AC30	IC2	2	Complete Genome	195	2	245.0	CP007577.1
NIPH 1669	IC3	4	Scaffold	106	3	139.0	APOQ01
UKK_0004	IC4	4	This study	438	15	113.0	ERS1042932
TG02011	IC5	1C	Contig	205	79	79.0	ASES01
BMBF-448	IC6	not done^a^	This study	944	78	76.0	ERS1047685
1429530	IC7	1A	Contig	229	25	113.3	JEWM01
LAC-4	IC8	4	Complete Genome	447	10	116.0	NZ_CP007712.1
NIPH 601	n/a	1B	Scaffold	373	40	86.0	APQZ01
AA-014	n/a	1D	Contig	499	158	63.9	AMGA01
6013150	n/a	1E	Scaffold	498	81	59.8	ACYQ02
268680	n/a	6	Contig	355	16	95.2	JEYN01

n/a: not available

^a^STs above ST920 were not available when BAPS analysis was performed, therefore not included in BAPS partitioning

### Evaluation and calibration of the cgMLST target gene set

To evaluate the newly defined cgMLST scheme, all available *A*. *baumannii* NCBI genome datasets (as of 2016-08-29) were downloaded, analyzed with the cgMLST and the ‘Oxford’ and ‘Pasteur’ MLST schemes, and filtered by ST. Also NCBI data that were used as reference or query genomes for scheme definition were removed. It was assumed that a suitable cgMLST scheme should reach on average at least 97.5% cgMLST called targets for all of those quality-filtered genomes. In addition, genome data for the three closely related species from the ACB complex, i.e. *A*. *nosocomialis*, *A*. *pittii*, and *A*. *calcoaceticus* were added for demonstration of the applicability of the defined cgMLST scheme for *A*. *baumannii* sensu stricto only.

To further calibrate the *A*. *baumannii* cgMLST scheme to investigate outbreaks, 53 sequenced carbapenem-resistant isolates were analysed using Ridom SeqSphere^+^ software to determine the presence of the target genes. Again we assumed that a well-defined core genome would cover at least 97.5% of the cgMLST genes used in this scheme. The target genes were extracted as previously described with “required identity to reference sequence of 90%”, “required aligned to reference sequence with 100%” [17] and the process included an assessment of the quality of the target genes, i.e. the absence of frame shifts and ambiguous nucleotides. A core genome gene was considered a “good target” only if all of the above criteria were met, in which case the complete sequence was analyzed in comparison to the ACICU reference. Alleles for each gene were called and assigned automatically by the SeqSphere^+^ software to ensure unique nomenclature. The combination of all alleles in each strain formed an allelic profile that was used to generate minimum spanning trees (MST) using the parameter “pairwise ignore missing values” during distance calculation. The MST was used to determine if outbreak isolates could be attributed to the same cluster and clearly separated from other clusters. To maintain backwards compatibility, classical MLST alleles (Oxford and Pasteur schemes) were extracted from the assembled genomes with SeqSphere^+^ using the PubMLST nomenclature (http://pubmlst.org/).

## Results

Species variability was checked by BAPS analysis based on 913 ‘Oxford‘ MLST ST which resulted in 8 partitions with ST 262 being the only member of BAPS partition 8. From the remaining 912 analysed STs, 312 showed significant admixture (*P*> 0.05) and were removed from the analysis, and 600 STs could be grouped into 7 partitions with a probability of ≥ 0.95 (S1 Table). Members of the largest partition 1 were located at five distinct branches of the ML tree, therefore this partition was further subdivided manually into five subgroups according to the branching of the tree (Partition 1A – 1E) (S1 Fig). For BAPS partitions 5 and 7 no strains, genomic data, or taxonomic information were available neither from NCBI or PubMLST. Strain data of BAPS partitions 3 and 8 were not considered for core genome genes definition because all known isolates of these groups were taxonomically identified as *A*. *nosocomialis*. Members from the remaining BAPS partitions and eight international clone lineages were included as query genomes for core genome definition (Table 2).

Based on these data the cgMLST Target Definer created a cgMLST scheme comprising 2,390 targets of the ACICU reference genome (58.6% of the complete genome) (S2 Table). A total of 1,573 *A*. *baumannii* datasets could be downloaded from NCBI Genomes and used. Genome data for which no MLST ST (‘Oxford’ and/or ‘Pasteur’) could be extracted were removed. Furthermore, the ST information was used to remove wrongly as *A*. *baumannii* identified genomes (e.g., *A*. *nosocomialis*). Thereby, in total 1,339 *A*. *baumannii* genomes were used to challenge the newly defined cgMLST scheme. On average 98.4% cgMLST genes were called successfully for those genomes. Analysis of datasets from the closely related species *A*. *nosocomialis*, *A*. *pittii*, and *A*. *calcoaceticus* resulted in 71.2%, 33.3%, and 23.2% good targets, respectively, thus demonstrating the applicability of the defined cgMLST scheme for *A*. *baumannii* sensu stricto only (S3 Table).

Fifty-three *A*. *baumannii* genomes were sequenced from isolates which were collected from patients hospitalized in nine hospitals located in eight German cities between 2005–2013. A summary of the genome assembly is shown in Table 1. Average coverage ranged from 37- to 124-fold, with a median of 102-fold. The number of contigs ranged from 67–333, with a median of 123. The percentage of good targets based on the core genome ranged from 98.7%−99.9% with a median of 99.3%.

**Fig 1:**
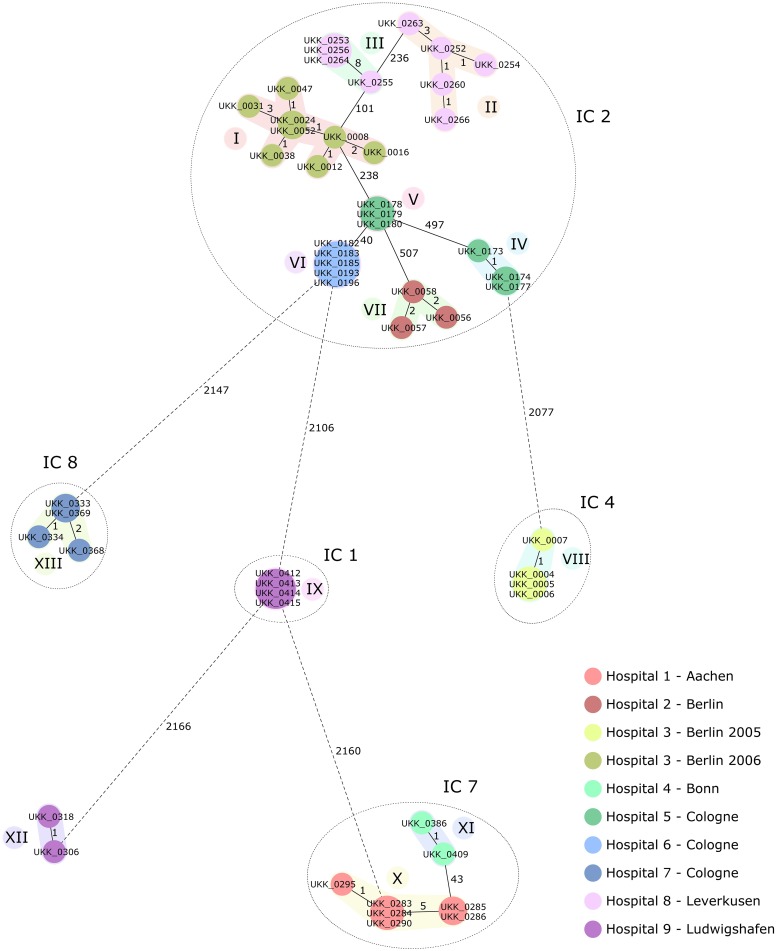
Minimum spanning tree based on cgMLST allelic profiles of 53 *A*. *baumannii* isolates. Each circle represents an allelic profile based on sequence analysis of 2,390 cgMLST target genes. The numbers on the connecting lines illustrate the numbers of target genes with different alleles. Colours of the circles represent the different isolation sources. Closely related genotypes (≤10 alleles difference) are shaded and clusters are numbered consecutively (I to XIII). Isolates belonging to the same IC lineage are surrounded by dotted circles.

There was a good correlation between PFGE types and cgMLST clusters. Based on their cgMLST profiles (S4 Table), a minimum spanning tree was generated (Fig 1). Fifty-three isolates were grouped into 13 distinct clusters (I to XIII, Table 1). Within a cluster, there were several examples where isolates were identical within the same hospital outbreak (clusters V, VI and IX), ≤ 5 differences (clusters I, II, IV, VII, VIII, XI, XII, XIII) and ≥ 5 differences (III andX,). Clusters IV and V contained isolates from the same hospital that were isolated within a 5-week period. However, although they shared the same Pasteur sequence type (ST2) and were double locus variants (DLV) using the Oxford scheme, they differed in 497 alleles. The number of allelic differences did not correlate with length of outbreak. For example isolate UKK_0255 from cluster III was collected on the same day as UKK_0253 and UKK_0256 but differed by 8 alleles, while cluster VI isolates were collected over a 5-week period and were identical.

The cgMLST clustering also matched the classification of international clones as previously determined by DiversiLab typing or MLST. The investigated outbreaks included strains that we determined to belong to IC1, IC2, IC4, IC7, IC8, and one set of strains that were not part of these clonal lineages (Fig 1). Clusters I-VII had up to 507 differences within IC2, but had >2000 differences to clusters belonging to other lineages.

## Discussion

For the thorough investigation of hospital outbreaks of bacterial infections, especially in the globalised society we now live in, simple, accurate and portable typing methods are essential. Thus, the ability to determine clonality among bacterial strains and to share this information in a centralised database has many advantages. MLST can be considered the proof of principle of a sequence-based method with a curated and standardised nomenclature [33]. However classical MLST does not have the resolution to determine person-person spread [16]. More discriminatory DNA fingerprinting methods such as PFGE and rep-PCR (DiversiLab) are not always comparable between different laboratories [15]. The use of WGS and user-friendly software means that whole genomes can be sequenced and compared within a few working days [17]. In our present study, we demonstrated that our *A*. *baumannii* cgMLST scheme was able to distinguish different outbreaks from the same hospital which shared the same or similar MLST profiles. An additional result was that we were clearly able to distinguish between different international *A*. *baumannii* clones.

Although there are now a plethora of sequenced *A*. *baumannii* genomes, few outbreaks have been investigated by WGS, and where applied to outbreak analysis, all the studies have performed SNV analysis, whereas our approach was allele-based [34–37]. In all of these cases, the SNV approach was compared to PFGE and was found to be generally in accordance with a few exceptions. For example, Salipante et al reported discrepancies between PFGE and WGS [36]. Halachev et al. applied SNV analysis during a prolonged *A*. *baumannii* outbreak and was able to differentiate between outbreak and non-outbreak strains and when combined with epidemiological data was able to reconstruct potential transmissions [37]. The ability to differentiate between different *A*. *baumannii* clonal lineages was also investigated and a threshold of 2,500 core SNPs was determined to accurately distinguish isolates from different clonal lineages [35]. In our study, we found that there were in the order of approximately 2000 allelic differences between different clonal lineages.

However, SNV analysis has to be carefully analysed and a common nomenclature may be difficult to adopt as there are multiple variations at the genome level both in ORFs and intergenic regions. Furthermore, during infection, several studies have shown that SNVs can develop rapidly and are often associated with antibiotic resistance determinants [38,39]. Indeed, Halachev et al. found several SNVs in *pmrB* which was associated with reduced susceptibility to colistin. Our cgMLST scheme does not follow the SNV approach, instead we have opted for an allele-based method. This has the advantage that it can “soften” the conflicting signals of horizontal-gene transfer such as a bias introduced by a single homologous recombination in a gene that typically results in multiple SNVs but only in single allele change. A further advantage of our allele-based typing is its relatively easy storage and curation in a centralised database, similar that seen with classical MLST [16,40]. To date, cgMLST schemes using the Ridom SeqSphere^+^ software have been established with *Listeria monocytogenes*, *Staphylococcus aureus*, *Legionella pneumophila*, *Francisella tularensis*, and *Mycobacterium tuberculosis* [17,41–44].

Bacterial genomes show high diversity and this makes the definition of a core-genome difficult. Previous work has shown that there are eight international *A*. *baumannii* clonal lineages. Of these, IC2 is the most prevalent clone worldwide and indeed is responsible for the majority of outbreaks [8,11]. Therefore, we took the approach that the core genome should be built around an IC2 isolate, which was why ACICU strain was chosen. From a genome of 3972 ORFs (ACICU genome), we determined that the core genome was made up of 2,390 genes. Based on the strains that we investigated, the number of missing targets was very small, the maximum was always below <2.5% of total targets. Some of these missing targets are caused by the presence of IS elements that insert into genes. These IS elements can be present in multiple copies and are one of the reasons for genome misassembly and multiple contigs [45].

In conclusion, we present a highly representative and discriminatory cgMLST scheme for WGS-based typing of *A*. *baumannii*. We were able to differentiate between strains obtained from patients at the same hospital and to link isolates from patients hospitalized in different cities. Easy to use software and public nomenclature will be the key for wide-spread adoption and contribution to outbreak analysis and general epidemiological surveillance.

## Supporting information

S1 Fig Partitions as determined by BAPS mapped on to a radial phylogram generated by FastTree 2. (A) Overview and (B) Zoom into the central part of the tree. BAPS partition 1 was further subdivided manually into five subgroups (1A-1E) according to the branching of the tree. STs that have significant admixture are colored in black.
(PDF)

S1 Table List of BAPS partitioning results per Oxford MLST sequence type. (XLSX)

S2 Table List of core genome genes used for the *A*. *baumannii* cgMLST scheme. (XLS)

S3 Table List of non-*Acinetobacter baumannii* genomes used for *A*. *baumannii* core genome genes evaluation. (XLSX)

S4 Table Allelic profiles of all 53 *A*. *baumannii* isolates used for cgMLST cluster type calibration. (XLSX)

## References

[1] Peleg AY, Seifert H, Paterson DL (2008) *Acinetobacter baumannii*: Emergence of a successful pathogen. Clin Microbiol Rev 21: 538–582. 10.1128/CMR.00058-07 18625687 PMC2493088

[2] Dijkshoorn L, Nemec A, Seifert H (2007) An increasing threat in hospitals: multidrug-resistant *Acinetobacter baumannii*. Nature Rev Microbiol 5: 939–951. 18007677 10.1038/nrmicro1789

[3] Wagenvoort JHT, De Brauwer EIGB, Toenbreker HMJ, van der Linden CJ (2002) Epidemic *Acinetobacter baumannii* strain with MRSA-like behaviour carried by healthcare staff. Eur J Clin Microbiol Infect Dis 21: 326–327. 10.1007/s10096-002-0716-2 12072949

[4] Husni RN, Goldstein LS, Arroliga AC, Hall GS, Fatica C, Stoller JK, et al (1999) Risk factors for an outbreak of multi-drug-resistant *Acinetobacter* nosocomial pneumonia among intubated patients. Chest 115: 1378–1382. 10334156 10.1378/chest.115.5.1378

[5] Chmielarczyk A, Higgins PG, Wojkowska-Mach J, Synowiec E, Zander E, Romaniszyn D, et al (2012) Control of an outbreak of *Acinetobacter baumannii* infections using vaporized hydrogen peroxide. J Hosp Infect 81: 239–245. 10.1016/j.jhin.2012.05.010 22727825

[6] Dijkshoorn L, Aucken H, Gerner Smidt P, Janssen P, Kaufmann ME, Garaizar J, et al (1996) Comparison of outbreak and nonoutbreak *Acinetobacter baumannii* strains by genotypic and phenotypic methods. J Clin Microbiol 34: 1519–1525. 8735109 10.1128/jcm.34.6.1519-1525.1996 PMC229053

[7] van Dessel H, Dijkshoorn L, van der Reijden T, Bakker N, Paauw A, van den Broek P, et al (2004) Identification of a new geographically widespread multiresistant *Acinetobacter baumannii* clone from European hospitals. Res Microbiol 155: 105–112. 10.1016/j.resmic.2003.10.003 14990262

[8] Higgins PG, Dammhayn C, Hackel M, Seifert H (2010) Global spread of carbapenem-resistant *Acinetobacter baumannii*. J Antimicrob Chemother 65: 233–238. 10.1093/jac/dkp428 19996144

[9] Diancourt L, Passet V, Nemec A, Dijkshoorn L, Brisse S (2010) The population structure of *Acinetobacter baumannii*: expanding multiresistant clones from an ancestral susceptible genetic pool. PloS ONE 5: e10034 10.1371/journal.pone.0010034 20383326 PMC2850921

[10] Higgins PG, Janßen K, Fresen MM, Wisplinghoff H, Seifert H (2012) Molecular epidemiology of *Acinetobacter baumannii* bloodstream isolates obtained in the United States from 1995 to 2004 using rep-PCR and multilocus sequence typing. J Clin Microbiol 50: 3493–3500. 10.1128/JCM.01759-12 22895032 PMC3486219

[11] Tomaschek F, Higgins PG, Stefanik D, Wisplinghoff H, Seifert H (2016) Head-to-head comparison of two multi-locus sequence typing (MLST) schemes for characterization of *Acinetobacter baumannii* outbreak and sporadic isolates. PloS ONE 11: e0153014 10.1371/journal.pone.0153014 27071077 PMC4829225

[12] Ribot EM, Fair MA, Gautom R, Cameron DN, Hunter SB, Swaminathan B, et al (2006) Standardization of pulsed-field gel electrophoresis protocols for the subtyping of *Escherichia coli* O157: H7 *Salmonella*, and *Shigella* for PulseNet. Foodborne Pathog Dis 3: 59–67. 10.1089/fpd.2006.3.59 16602980

[13] Murchan S, Kaufmann ME, Deplano A, de Ryck R, Struelens M, Zinn CE, et al (2003) Harmonization of pulsed-field gel electrophoresis protocols for epidemiological typing of strains of methicillin-resistant *Staphylococcus aureus*: a single approach developed by consensus in 10 European laboratories and its application for tracing the spread of related strains. J Clin Microbiol 41: 1574–1585. 10.1128/JCM.41.4.1574-1585.2003 12682148 PMC153895

[14] Seifert H, Dolzani L, Bressan R, van der Reijden T, van Strijen B, Stefanik D, et al (2005) Standardization and interlaboratory reproducibility assessment of pulsed-field gel electrophoresis-generated fingerprints of *Acinetobacter baumannii*. J Clin Microbiol 43: 4328–4335. 10.1128/JCM.43.9.4328-4335.2005 16145073 PMC1234071

[15] Higgins PG, Hujer AM, Hujer KM, Bonomo RA, Seifert H (2012) Interlaboratory reproducibility of DiversiLab rep-PCR typing and clustering of *Acinetobacter baumannii* isolates. J Med Microbiol 61: 137–141. 10.1099/jmm.0.036046-0 21903821 PMC3347881

[16] Perez-Losada M, Cabezas P, Castro-Nallar E, Crandall KA (2013) Pathogen typing in the genomics era: MLST and the future of molecular epidemiology. Infecta Genet Evol 16: 38–53. 10.1016/j.meegid.2013.01.009 23357583

[17] Ruppitsch W, Pietzka A, Prior K, Bletz S, Fernandez HL, Allerberger F, et al (2015) Defining and evaluating a core genome multilocus sequence typing scheme for whole-genome sequence-based typing of *Listeria monocytogenes*. J Clin Microbiol 53: 2869–2876. 10.1128/JCM.01193-15 26135865 PMC4540939

[18] Eyre DW, Golubchik T, Gordon N, Bowden R, Piazza P, Batty EM, et al (2012) A pilot study of rapid benchtop sequencing of *Staphylococcus aureus* and *Clostridium difficile* for outbreak detection and surveillance. BMJ Open 2: e001124 10.1136/bmjopen-2012-001124 22674929 PMC3378946

[19] Mellmann A, Harmsen D, Cummings CA, Zentz EB, Leopold SR, Rico A, et al (2011) Prospective genomic characterization of the German Enterohemorrhagic *Escherichia coli* O104:H4 outbreak by rapid next generation sequencing technology. PloS ONE 6: e22751 10.1371/journal.pone.0022751 21799941 PMC3140518

[20] Turabelidze G, Lawrence SJ, Gao H, Sodergren E, Weinstock GM, Abubucker S, et al (2013) Precise dissection of an *Escherichia coli* O157:H7 outbreak by single nucleotide polymorphism analysis. J Clin Microbiol 51: 3950–3954. 10.1128/JCM.01930-13 24048526 PMC3838074

[21] Wyres KL, Conway TC, Garg S, Queiroz C, Reumann M, Holt K, et al (2014) WGS analysis and interpretation in clinical and public health microbiology laboratories: what are the requirements and how do existing tools compare? Pathogens 3: 437–458. 10.3390/pathogens3020437 25437808 PMC4243455

[22] Fricke W, Rasko DA (2014) Bacterial genome sequencing in the clinic: bioinformatic challenges and solutions. Nat Rev Genet 15: 49–55. 10.1038/nrg3624 24281148

[23] Kohlenberg A, Brummer S, Higgins PG, Sohr D, Piening BC, de Grahl C, et al (2009) Outbreak of carbapenem-resistant Acinetobacter baumannii carrying the carbapenemase OXA-23 in a German University Medical Centre. J Med Microbiol 58: 1499–1507. 10.1099/jmm.0.012302-0 19589905

[24] Molter G, Seifert H, Mandraka F, Kasper G, Weidmann B, Hornei B, et al (2016) Outbreak of carbapenem-resistant *Acinetobacter baumannii* in the intensive care unit: a multi-level strategic management approach. J Hospital Infect 92: 194–198. 10.1016/j.jhin.2015.11.007 26778130

[25] Juenemann S, Sedlazeck FJ, Prior K, Albersmeier A, John U, Kalinowski J, et al (2013) Updating benchtop sequencing performance comparison. Nat Biotech 31: 294–296. 10.1038/nbt.2522 23563421

[26] Zerbino DR, Birney E (2008) Velvet: Algorithms for de novo short read assembly using de Bruijn graphs. Genome Res 18: 821–829. 10.1101/gr.074492.107 18349386 PMC2336801

[27] Corander J, Marttinen P, Siren J, Tang J (2008) Enhanced Bayesian modelling in BAPS software for learning genetic structures of populations. BMC Bioinformatics 9: 539 10.1186/1471-2105-9-539 19087322 PMC2629778

[28] Bartual SG, Seifert H, Hippler C, Luzon MAD, Wisplinghoff H, Rodriguez-Valera F (2005) Development of a multilocus sequence typing scheme for characterization of clinical isolates of *Acinetobacter baumannii*. J Clin Microbiol 43: 4382–4390. 10.1128/JCM.43.9.4382-4390.2005 16145081 PMC1234098

[29] Edgar RC (2004) MUSCLE: multiple sequence alignment with high accuracy and high throughput. Nucl Acids Res 32: 1792–1797. 10.1093/nar/gkh340 15034147 PMC390337

[30] Price MN, Dehal PS, Arkin AP (2010) FastTree 2-Approximately Maximum-Likelihood Trees for Large Alignments. PloS ONE 5: e9490 10.1371/journal.pone.0009490 20224823 PMC2835736

[31] Huson DH, Richter DC, Rausch C, Dezulian T, Franz M, Rupp R (2007) Dendroscope: An interactive viewer for large phylogenetic trees. BMC Bioinformatics 8: 460 10.1186/1471-2105-8-460 18034891 PMC2216043

[32] Iacono M, Villa L, Fortini D, Bordoni R, Imperi F, Bonnal RJP, et al (2008) Whole-genome pyrosequencing of an epidemic multidrug-resistant *Acinetobacter baumannii* strain belonging to the European clone II group. Antimicrob Agents Chemother 52: 2616–2625. 10.1128/AAC.01643-07 18411315 PMC2443898

[33] Maiden MCJ, Bygraves JA, Feil E, Morelli G, Russell JE, Urwin R, et al (1998) Multilocus sequence typing: A portable approach to the identification of clones within populations of pathogenic microorganisms. Proc Natl Acad Sci USA 95: 3140–3145. 9501229 10.1073/pnas.95.6.3140 PMC19708

[34] Hammerum AM, Hansen F, Skov MN, Stegger M, Andersen PS, Holm A, et al (2015) Investigation of a possible outbreak of carbapenem-resistant *Acinetobacter baumannii* in Odense, Denmark using PFGE, MLST and whole-genome-based SNPs. J Antimicrob Chemother 70: 1965–1968. 10.1093/jac/dkv072 25795772

[35] Fitzpatrick MA, Ozer EA, Hauser AR (2016) Utility of whole-genome sequencing in characterizing *Acinetobacter* epidemiology and analyzing hospital outbreaks. J Clin Microbiol 54: 593–612. 10.1128/JCM.01818-15 26699703 PMC4767972

[36] Salipante SJ, SenGupta DJ, Cummings LA, Land TA, Hoogestraat DR, Cookson BT (2015) Application of whole-genome sequencing for bacterial strain typing in molecular epidemiology. J Clin Microbiol 53: 1072–1079. 10.1128/JCM.03385-14 25631811 PMC4365209

[37] Halachev M, Chan J, Constantinidou C, Cumley N, Bradley C, Smith-Banks M, et al (2014) Genomic epidemiology of a protracted hospital outbreak caused by multidrug-resistant *Acinetobacter baumannii* in Birmingham, England. Genome Med 6: 70 10.1186/s13073-014-0070-x 25414729 PMC4237759

[38] Hornsey M, Loman N, Wareham DW, Ellington MJ, Pallen MJ, Turton JF, et al (2011) Whole-genome comparison of two *Acinetobacter baumannii* isolates from a single patient, where resistance developed during tigecycline therapy. J Antimicrob Chemother 66: 1499–1503. 10.1093/jac/dkr168 21565804

[39] Higgins PG, Schneiders T, Hamprecht A, Seifert H (2010) In vivo selection of a missense mutation in adeR and conversion of the novel *bla*_OXA-164_ into *bla*_OXA-58_ in carbapenem-resistant *Acinetobacter baumannii* isolated from a hospitalized patient. Antimicrob Agents Chemother 54: 5021–5027. 10.1128/AAC.00598-10 20921306 PMC2981280

[40] Maiden MC, van Rensburg MJ, Bray JE, Earle SG, Ford SA, Jolley KA, et al (2013) MLST revisited: the gene-by-gene approach to bacterial genomics. Nat Rev Microbiol 11: 728–736. 10.1038/nrmicro3093 23979428 PMC3980634

[41] Kohl TA, Diel R, Harmsen D, Rothgaenger J, Walter KM, Merker M, et al (2014) Whole-genome-based *Mycobacterium tuberculosis* surveillance: a standardized, portable, and expandable approach. J Clin Microbiol 52: 2479–2486. 10.1128/JCM.00567-14 24789177 PMC4097744

[42] Moran-Gilad J, Prior K, Yakunin E, Harrison TG, Underwood A, Lazarovitch T, et al (2015) Design and application of a core genome multilocus sequence typing scheme for investigation of Legionnaires' disease incidents. Eurosurveillance 20: 21087. 26212142 10.2807/1560-7917.es2015.20.28.21186

[43] Antwerpen MH, Prior K, Mellmann A, Hoppner S, Splettstoesser WD, Harmsen D (2015) Rapid high resolution genotyping of *Francisella tularensis* by whole genome sequence comparison of annotated genes ("MLST+"). PloS ONE 10. 10.1371/journal.pone.0123298 PMC4391923 25856198

[44] Leopold SR, Goering RV, Witten A, Harmsen D, Mellmann A (2014) Bacterial whole-genome sequencing revisited: Portable, scalable, and standardized analysis for typing and detection of virulence and antibiotic resistance genes. J Clin Microbiol 52: 2365–2370. 10.1128/JCM.00262-14 24759713 PMC4097726

[45] Adams MD, Goglin K, Molyneaux N, Hujer KM, Lavender H, Jamison JJ, et al (2008) Comparative genome sequence analysis of multidrug-resistant *Acinetobacter baumannii*. J Bacteriol 190: 8053–8064. 10.1128/JB.00834-08 18931120 PMC2593238

