# Supplementary material for: Development and evaluation of a core genome multilocus typing scheme for whole-genome sequence-based typing of Acinetobacter baumannii
Source: PLoS One. 2017 Jun 8;12(6):e0179228. doi: 10.1371/journal.pone.0179228 (PMC5464626; doi:10.1371/journal.pone.0179228)
Supplement: S1 Fig — (A) Overview and (B) Zoom into the central part of the tree. BAPS partition 1 was further subdivided manually into five subgroups (1A-1E) according to the branching of the tree. STs that have significant admixture are colored in black. (PDF) [file pone.0179228.s001.pdf]

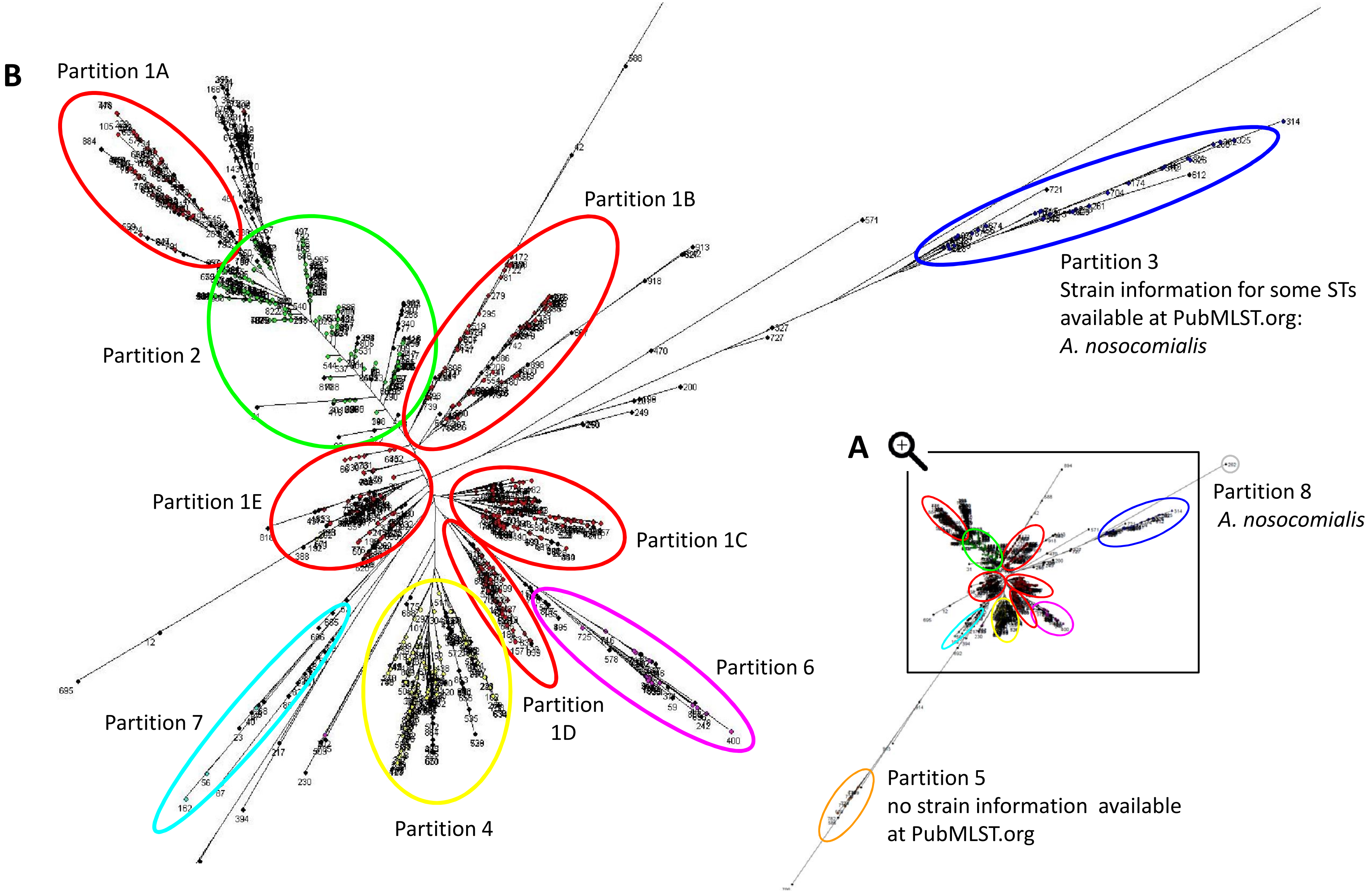

Supp. Fig.1: Partitions as determined by BAPS mapped onto a radial phylogram generated by FastTree 2. (A) Overview and (B) Zoom into the central part of the tree. BAPS partition 1 was further subdivided manually into five subgroups (1A - 1E) according to the branching of the tree. STs that have significant admixture are colored in black.
